# Alpha-Synuclein Phosphomimetic Y39E and S129D Knock-In Mice Show Cytosolic Alpha-Synuclein Localization without Developing Neurodegeneration or Motor Deficits

**DOI:** 10.1523/ENEURO.0357-24.2025

**Published:** 2025-04-10

**Authors:** YoungDoo Kim, Bhupesh Vaidya, Joseph McInnes, Huda Y. Zoghbi

**Affiliations:** ^1^Department of Molecular and Human Genetics, Baylor College of Medicine (BCM), Houston, Texas 77030; ^2^Jan and Dan Duncan Neurological Research Institute at Texas Children’s Hospital, Houston, Texas 77030; ^3^Department of Neuroscience, BCM, Houston, Texas 77030; ^4^Department of Pediatrics, BCM, Houston, Texas 77030; ^5^Department of Neurology, BCM, Houston, Texas 77030; ^6^Howard Hughes Medical Institute, Houston, Texas 77030

**Keywords:** alpha-synuclein, phosphomimetic mutants, S129D, Y39E

## Abstract

Parkinson's disease (PD) is a progressive neurodegenerative disorder characterized by motor and nonmotor symptoms. Its pathological hallmarks include the accumulation of misfolded alpha-synuclein (α-Syn) in Lewy bodies and Lewy neurites. Phosphorylation of α-Syn is a prominent feature of these inclusions, but its role in disease pathogenesis remains unclear. To identify the role of α-Syn phosphorylation in synucleinopathy, we generated two *Snca* knock-in (KI) mouse models carrying phosphomimetic mutations at SncaY39 or SncaS129 (*Snca^Y39E^* or *Snca^S129D^*) which manipulated epitopes phosphorylated in the PD brain. Both *Snca^Y39E^* and *Snca^S129D^* KI mice displayed increased α-Syn phosphorylation, enhanced oligomer formation, and a shift of α-Syn localization from membrane-bound to cytoplasm. However, neurodegeneration in the substantia nigra was not observed up to 24 months of age. These findings demonstrate that mimicking the phosphorylation of Y39 or S129 can induce endogenous α-Syn phosphorylation. Still, a single phosphomimetic mutation alone is insufficient to induce PD-like behavior and pathology in the mouse's lifespan. Overall, our study provides a mouse model for investigating the role of phosphorylation at Y39 and S129 α-Syn epitopes in vivo.

## Significance Statement

Phosphorylation of specific alpha-synuclein (α-Syn) epitopes is observed in Parkinson’s disease and other Lewy body diseases, but the direct relationship between phosphorylation at these sites and disease pathology remains unclear. This study focuses on two epitopes Y39 and S129, known to induce α-Syn protein aggregation in vitro, but in vivo data are either controversial (S129D) or missing (Y39E). We generated two phosphomimetic mutant (*Snca^Y39E^* and *Snca^S129D^*) KI mice that show shifting of α-Syn localization from the membrane to the cytosol and enhancement of protein oligomerization, highlighting a potential role of these α-Syn phosphorylation sites in the initial steps of protein aggregation.

## Introduction

In Parkinson’s disease (PD) and dementia with Lewy body patient’s brain, neurons contain intracellular dense protein aggregates known as Lewy bodies (Baba et al., 1998, Braak et al., 2003). The alpha-synuclein (α-Syn) phosphorylation is the hallmark of these aggregates (Okochi et al., 2000). Among the multiple phosphorylation sites on α-Syn, serine 129 (S129) was first identified as phosphorylated in PD patients and remains a key marker for α-Syn pathology (Anderson et al., 2006). Besides S129, among other serine and tyrosine residues, tyrosine 39 (Y39) is phosphorylated and detected in Lewy bodies (Brahmachari et al., 2016), and Y39 phosphorylation increased the fibrilization of α-Syn in vitro (Zhao et al., 2020). Y39 is the only phosphorylation site within the amphipathic domain (core of membrane-binding domain) of α-Syn, where all human PD mutations have been reported (Kawahata et al., 2022).

The mechanism of how α-Syn forms aggregates is still being debated, but one hypothesis proposes that phosphorylated α-Syn detaches from the membrane, localizes to the cytosol, and forms aggregates (Pineda and Burré 2017). In line with this, it has been reported that familial PD-causing mutations of α-Syn, such as A30P, G51D, and A53E, show reduced membrane binding of α-Syn protein (Snead and Eliezer 2014). Moreover, phosphorylation of α-Syn at Y39 and S129 decreases its membrane-binding affinity (Fiske et al., 2011, Dikiy et al., 2016). Although there is a linkage between α-Syn phosphorylation and pathologies of synucleinopathy, the direct contribution of phosphorylation to the pathology remains unclear.

Several in vitro and invertebrate studies of phosphomimetic mutations (Y39E or S129D) showed that these mutations accelerated α-Syn aggregation with reduced membrane binding, highlighting their role in α-Syn's mislocalization and aggregation (Chen and Feany 2005, Kuwahara et al., 2012, Dikiy et al., 2016). For mouse model studies, S129 phosphomimetic (S129D) or phospho-incompetent (S129A) α-Syn viral overexpression (Gorbatyuk et al., 2008, McFarland et al., 2009) or transgenic mice (Escobar et al., 2014) have been used to elucidate the role of phosphorylation at serine 129 of α-Syn in the pathology of PD and other synucleinopathies. However, these models failed to produce mutant-specific phenotypes even though each mutation results in the reverse phosphorylation status. For instance, relatively high expression of either S129A or S129D α-Syn by viral vector led to the degeneration of dopaminergic neurons regardless of mutation types (Gorbatyuk et al., 2008), and transgenic mice showed relatively low expression of S129A or S129D mutants; neither of them showed neuronal degeneration (Escobar et al., 2014). These data suggest that current mouse models of phospho-mimetic or phospho-incompetent α-Syn exhibit its toxicity due to the elevated protein expression levels, but not their phosphorylation status. Also, no *Snca^Y39E^* mouse model is available to study the in vivo role of α-Syn Y39 phosphorylation. Therefore, to better understand the pathological contribution of site-specific phosphorylation of α-Syn protein, it is critical to generate a mouse model that accurately recapitulates the phosphorylation changes in the relevant brain tissues while maintaining an endogenous expression level of wild-type (WT) or mutant α-Syn.

To investigate the effect of α-Syn phosphorylation in a physiologically relevant context, we used CRISPR-Cas9 to edit the endogenous mouse *Snca* gene and develop phosphomimetic α-Syn knock-in (KI) mouse models containing single phosphorylation sites, Y39E and S129D, which are hyperphosphorylated in PD patients. In both KI lines, we found that α-Syn exhibited reduced lipid membrane binding, increased cytosolic localization, and promoted the formation of soluble oligomers. However, these changes did not result in neuroinflammation, and the mice did not exhibit motor deficits up to 12 months of age and no dopaminergic neuron degeneration up to 24 months. These findings suggest that the Snca^Y39E^ and Snca^S129D^ KI lines are valuable in vivo tools for studying α-Syn protein localization changes and oligomerization with Y39 or S129 phosphorylation.

## Material and methods

### Animals

All mice were housed in a Level 3, American Association for Laboratory Animal Science (AALAS)-certified facility on a 14 h light/dark cycle. All animal procedures were performed in accordance with the Baylor College of Medicine animal care committee’s regulations.

### Generation of α-Syn familial mutant KI mice

*Snca^Y39E^* and *Snca^S129D^* KI mice were generated via CRISPR-CAS9–mediated standard homologous recombination methods in the Genetically Engineered Rodent Models Core (Baylor College of Medicine). Mice were bred in C57BL/6J background. We designed crRNA 20 nucleotides upstream of the PAM site to a region of interest using crispr.mit.edu. To design ssODN for point-mutation KI, we inserted a point mutation flanked by 6–8 synonymous mutations (ideally AT to GC to get a high Tm for genotyping), followed by flanking with a 75-nucleotide left homology arm and a 100-nucleotide right homology arm. This is designed to destroy the PAM or gRNA sequence with synonymous mutations to prevent recutting and to allow for genotyping by differential primer hybridization. We mixed 40 ng of crRNA with 40 ng Alt-R CRISPR-Cas9 tracrRNA, 1 µg of ssODN, and 30 ng of Alt-R S.p. HiFi Cas9 Nuclease V3 in 100 µl of T_10_E_0.1_ buffer before injection into mouse embryos. All the reagents were ordered from Integrated DNA Technologies. KI mice were confirmed by genomic PCR with different primers between the unmodified (WT) and modified (Y39E and S129D) alleles (for the sequences, Table 1).

**Table 1.** Materials used for this study

Reagent or resource	Source	Identifier
Antibodies		
Anti-α-Syn antibody (clone 42)	BD	RRID: AB_398107
p-S129-α-Syn (clone EP1536Y)	Abcam	RRID: AB_869973
p-S129-α-Syn (clone D1R1R)	Cell Signaling Technology	RRID: AB_279886
Anti-vinculin	Sigma-Aldrich	RRID: AB_477629
Anti-PSD95 (clone 7E3)	Cell Signaling Technology	RRID: AB_2721262
Anti-GFAP	Novus Biologicals	RRID: AB_829022
Anti-Iba1	Wako Chemicals	RRID: AB_839504
Anti-TH	EMD Millipore	RRID: AB_390204
Alexa Fluor 488 AffiniPure Donkey Anti-Mouse IgG (H + L)	Jackson ImmunoResearch Laboratories	RRID: AB_2340846
Donkey Anti-Rabbit IgG (H + L) Highly Cross-Adsorbed Secondary Antibody, Alexa Fluor 555	Thermo Fisher Scientific	RRID: AB_162543
Donkey Anti-Goat IgG (H + L) Cross-Adsorbed Secondary Antibody, Alexa Fluor 488	Thermo Fisher Scientific	RRID: AB_2534102
Chemical		
1× protease inhibitor cocktails	GenDEPOT	Catalog #P3100–100
1× phosphatase inhibitor cocktails	GenDEPOT	Catalog #P3200–020
Laemmli sample buffer	Sigma-Aldrich	Catalog #S3401–10VL
Amersham ECL Prime Western Blotting Detection Reagent	Cytiva	Catalog #RPN2236
Kit		
VECTASTAIN(R) ELITE(R) ABC Anti-Rabbit IgG HRP Immunodetection Kit	Vector Laboratories	Catalog #PK-6101
NativePage Sample kit	Thermo Fisher Scientific	Catalog #BN2008
Experimental models: Organisms/strain		
Mouse: wild type (WT: C57BL/6J)	Jackson Laboratory	Catalog #000664
Mouse: *Snca^Y39E^* KI	This paper	N/A
Mouse: *Snca^S129D^* KI	This paper	N/A
CRISPR-Cas9 reagents		
Alt-R CRISPR-Cas9 tracrRNA	Integrated DNA Technologies	Catalog #1072532
Alt-R S.p. HiFi Cas9 Nuclease V3	Integrated DNA Technologies	Catalog #1081060
Oligonucleotides		
crRNA for *Snca^Y39E^*: 5′-GGCAGCTGGAAAGACAAAAG-3′	Integrated DNA Technologies	N/A
crRNA for *Snca^S129D^*: 5′-GGCTTATGAAATGCCTTCAG-3′	Integrated DNA Technologies	N/A
ssODN for *Snca^Y39E^*: 5′-TTCAAAGGCCAAGGAGGGAGTTGTGGCTGCTG CTGAGAAAACCAAGCAGGGTGTGGCAGAGGCAGCTGGAAAGACGAAG GAAGGCGTGCTGGAAGTAGGTAGGTAGTGACACTGTGACTAATGAATT GGGGTGGCTGGTGTGTGGTGTCTGATTCGTGTGCATCACAGCTTCTCA GAAGAGTGACAGCTGTGTG-3′	Integrated DNA Technologies	N/A
ssODN for *Snca^S129D^*: 5′-GAGGAGGGGTACCCACAGGAAGGAATCCTGG AAGACATGCCTGTGGATCCTGGCAGCGAAGCCTACGAGATGCCGGAC GAAGTAAATGCCTGTATAAAGAAAACTAAGCAAAACACTTTAGGTGTTTA ATTTGGAACACATACCATCAAAACCCTGCCACTATCAGATCTCTCTCACA-3′	Integrated DNA Technologies	N/A
Genotyping primer for *Snca^Y39E^* and WT forward: anneals at 59° 5′-CCTCC CCTTCTGCAACTCTTCTTCTG-3′	Sigma-Aldrich	N/A
Genotyping primer for *Snca^Y39E^* reverse: anneals at 59° 5′-CTTCCAGCACG CCTTCCTTCG-3′	Sigma-Aldrich	N/A
Genotyping primer for WT sequence of *Snca^Y39E^* KI reverse: anneals at 59° 5′-GTCACTACCTACCTACATAGAGGACTCCCTCTTTTG-3′	Sigma-Aldrich	N/A
Genotyping primer for *Snca^S129D^* and WT forward: anneals at 63° 5′-GCCCT CATTATTCACCACACATGCACATAGTCCAC-3′	Sigma-Aldrich	N/A
Genotyping primer for WT sequence of *Snca^S129D^* KI reverse: anneals at 63° 5′-CTCTGAAGGCATTTCATAAGCCTCACT-3′	Sigma-Aldrich	N/A
Genotyping primer for *Snca^S129D^* reverse: anneals at 63° 5′-TTCGTCCGG CATCTCGTAGGCTTCGCT-3′	Sigma-Aldrich	N/A
Genomic sequencing primer for *Snca^Y39E^* forward: 5′-CAACAATCAAT CAACACTGTGCC-3′	Sigma-Aldrich	N/A
Genomic sequencing primer for *Snca^Y39E^* reverse: 5′-GTCTTTGATTCATAA GCATATTCTTGG-3′	Sigma-Aldrich	N/A
Genomic sequencing primer for *Snca^S129D^* forward: 5′-TCATTGAAAT GCTATGTGGGTTCTGTCTAC-3′	Sigma-Aldrich	N/A
Genomic sequencing primer for *Snca^S129D^* reverse: 5′-ACAAAATCCAGAT AACATTCATGACAGTAA-3′	Sigma-Aldrich	N/A
qPCR primer for *mSnca* forward: 5′-GTGACAACAGTGGCTGAGAAGAC-3′	Sigma-Aldrich	N/A
qPCR primer for *mSnca* reverse: 5′-GGTACCCCTCCTCACCCTTG-3′	Sigma-Aldrich	N/A
qPCR primer for *mGapdh* forward: 5′-AGGTCGGTGTGAACGGATTTG-3′	Sigma-Aldrich	N/A
qPCR primer for *mGapdh* reverse: 5′-TGTAGACCATGTAGTTGAGGTCA-3′	Sigma-Aldrich	N/A

### Behavioral assays

We tested 9- and 12-month-old heterozygous *Snca^Y39E^* and *Snca^S129D^* mice with their WT littermates. A series of behavioral tests were performed as a part of this study. Mice were acclimated to the test environments for 30 min before testing, with at least a 30 min interval between each trial.

#### Open-field test

The open-field arena is a 40 × 40 × 40 cm (width × length × height) cubical enclosure. The central 20 × 20 cm region of the box was marked by the ANY-maze software (Stoelting). The mouse was placed in a corner of the box and recorded by an overhead video camera for 10 min. Several measures were analyzed, including total distance traveled, mean motor speed, and center/total ratio.

#### Pole test

The mice were placed head up near the top of a vertical pole (threaded rod, 50 cm long, 1 cm in diameter), and the test lasted for 60 s. We counted the number of times the mouse turned their head and whole body downward (turn) and climbed down to the ground. The mice underwent three trials, and the minimum durations of “turn” and “down” time measured and “down” time were used for analysis.

#### Grip strength

A mouse was picked up from the base of a tail and gently lowered toward the net until it grasped the bar of the grip strength meter (Columbus Instruments 0167–8001). The mouse was then gently pulled backward until it released its grip. The maximum pull force at the time the animal released the grip was recorded on a horizontally mounted scale equipped with a drag pointer. Mice underwent three trials, and the maximum score across the three trials was used for the analysis.

#### Parallel rod floor test

Animals were placed individually into the center of a wire grid laid within an open-field chamber (AccuScan) for 10 min. The number of foot slips through the wire grid was recorded and analyzed using ANY-maze (Stoelting). The number of foot slips was normalized to the total distance traveled.

### Whole protein extraction

Mice were killed by isoflurane inhalation at 12 months of age. The midbrain region containing the substantia nigra was harvested and immediately frozen on dry ice. Samples were mixed with 5–10 volumes of modified RIPA buffer [50 mM Tris–Cl, 150 mM NaCl, 1% NP-40, 0.5% sodium deoxycholate, 0.1% sodium dodecyl sulfate (SDS)] supplemented with 0.5% Triton X-100, 1× protease inhibitor, and 1× phosphatase inhibitor buffer and lyzed with sonication step (20 pulses, output 2.5, duty cycle 30%, 2 and 2 s rest intervals, five times). Samples were centrifuged at 20,000 × *g* for 20 min, and the supernatant was collected for use.

### Sequential tissue extraction

To separate cytosol, membrane, and detergent-insoluble fractions, we modified the protocol from the previous study (Nuber et al., 2024). The 12-month-old WT, *Snca^Y39E/+^*, and *Snca^S129D/+^* mice midbrain tissues were lysed by adding 300 μl of TBS-EDTA buffer [50 mM Tris–HCl, 175 mM NaCl; 5 mM EDTA; protease inhibitor cocktail (GenDEPOT)], pH 7.4, and ultracentrifuged for 30 min at 120,000 × *g*. Supernatant is a cytosol-enriched fraction. The pellets were extracted in TBS-EDTA with 1% Triton X-100 and ultracentrifuged for 30 min at 120,000 × *g*. Supernatants are membrane-enriched fractions. Pellets were lyzed with TBS-EDTA buffer with 2% SDS for extract detergent-insoluble fraction. Sample loading was determined based on BCA quantification of the membrane-enriched fraction and further normalized to vinculin protein levels. To maintain consistency, we kept uniform the loading ratio between fractions for each sample.

### SDS–PAGE and Western blot

Protein samples were loaded on either 10- or 15-well NuPAGE 4–12% Bis–Tris gels (Thermo Fisher Scientific). Gels were run in 1× MES/SDS protein running buffer and transferred onto nitrocellulose membranes in Tris–glycine buffer (25 mM Tris, 190 mM glycine) supplemented with 10% methanol at 0.3 amps for 1.5 h. After being transferred, membranes were blocked in 5% milk in TBS-T for 1 h and probed with one of the following primary antibodies overnight: mouse anti-vinculin (1:10,000), mouse anti-α-Syn (1:3,000), rabbit anti-pS129-α-Syn (1:3,000), and mouse anti-pY39-α-Syn (1:3,000). Membranes were washed three times in TBS-T for 10 min, and secondary mouse or rabbit HRP-conjugated secondary antibodies were applied in 5% skim milk in TBS-T. Following the wash, ECL-induced chemiluminescence (Cytiva, RPN2236) was imaged by Amersham imager 680 (GE Healthcare).

### Native gel Western blot

For native-PAGE experiments, 12-month-old WT, *Snca^Y39E/+^*, and *Snca^S129D/+^* mice whole brains were lysed following the instructions of the NativePage Sample kit (Thermo Fisher Scientific, BN2008). Samples are homogenized with 1× NativePage sample buffer supplemented with 1% DDM and centrifuged in 20,000 × *g* for 20 min. Supernatants were collected and loaded without boiling and directly onto Native-PAGE 4–16% Bis-tri protein gels (Thermo Fisher Scientific, BN1004). After transfer to the membrane, Western blots were developed as described above.

### Immunofluorescence (IF) and immunohistochemistry (IHC)

#### Tissue preparation

For IF and IHC experiments, mice were transcranially perfused with PBS followed by 4% paraformaldehyde (PFA). Brains were dissected and fixed in 4% PFA for 2 d, dehydrated for 24 h in 15% sucrose (w/v, in PBS) followed by a 2 d incubation in 30% sucrose solution (in PBS), all at 4°C. The brains were then frozen on dry ice in OCT compound (VWR, 25608-930) and sectioned on a cryostat (Leica CM 3050S). Sections were collected at 40 µm thickness. Sections were kept in 1× PBS with 0.01% NaN_3_ until ready for use.

#### IF

IF was performed as previously described (Kim et al., 2023). Briefly, floating sections were washed three times in 1× PBS and then incubated with one or two of the following antibodies overnight: mouse anti-α-Syn (1:1,000), rabbit anti-Iba1 (1:1,000), goat anti-GFAP (1:1,000), and rabbit anti-tyrosine hydroxylase (TH; 1:1,000). Afterward, the sections were washed three more times and stained with secondary antibodies with a designated fluorophore (488 or 555 nm) with 1:1,000 concentrations in room temperature for 3 h. After three washings, the sections were incubated for 10 min with DAPI (Thermo Fisher Scientific, D1306, 1:200,000) and mounted with VECTASHIELD HardSet Antifade mounting media (Vector Laboratories, H-1400-10). Imaging was performed on a confocal microscope (Leica STED TCS SP8X), using the LAS X software (Leica) after selecting optimal settings for image capture. All IF images presented in this study were taken with the 63× objective lens.

#### IHC

Floating sections were washed three times in 1× PBS. The sections were then blocked with PBS supplemented with 5% normal goat serum and 0.3% Triton X-100 for 1 h and incubated with antibodies listed below overnight at 4°C: rabbit anti-pSerS129-α-Syn antibody (1:1,000) and rabbit anti-TH antibody (1:1,000). Afterward, the sections were washed three more times and stained with the VECTASTAIN Elite ABC HRP Kit (peroxidase, rabbit IgG) according to manufacturer’s instructions. Thereafter, DAB Peroxidase (HRP) Substrate Kit, 3,3′-diaminobenzidine, was used to develop the sections. Sections were mounted on SuperFrost plus slides (Thermo Fisher Scientific, 22-037-246) and dried at room temperature. Slides were then dehydrated by incubating them in the following series of solutions, PBS, H_2_O, 70% ethanol, 95% ethanol, 100% ethanol, and xylene, before mounting coverslips using Richard-Allan Scientific Cytoseal XYL (Thermo Fisher Scientific, 8312-4).

### Counting of dopaminergic neurons and neurites

Stereological counts were performed as previously described (Vázquez-Vélez et al., 2020). Briefly, sections from −2.54 to −4 mm from the bregma (for dopaminergic neurons in the substantia nigra) and +0.26 to −0.22 mm from the bregma (for dopaminergic neuron cell bodies in the striatum) were selected for staining in intervals of six. IHC for TH was performed as described above. All IHC for stereology was imaged using a 4× objective on a Ti2E spinning disk confocal microscope (Nikon). The experimenter outlined the substantia nigra or striatum and counted TH-positive cell bodies (SNc) or intensities (STR). The total number of cells was estimated using the measured tissue thickness (6–8 slices per brain region). To analyze the ratio of colocalization of TH with cytosolic α-Syn, we counted 20 to 40 neurons positive for both α-Syn and TH in each slice (200 to 300 per mouse). The number of TH^+^ neurons and the density of TH intensity were averaged for each animal.

### Statistical analysis

For all experiments, comparisons of two groups were performed using Student’s *t* test and unequal variance *t* test when the group sizes were very different (Fig. 6). Comparisons of three or more groups were performed using one-way ANOVA followed by Dunnett’s multiple-comparison test. All analyses were conducted using the Prism 10 software (GraphPad).

## Result

### Generation of *Snca* Y39E and S129D phosphomimetic mutant KI mice

**Figure 1.**
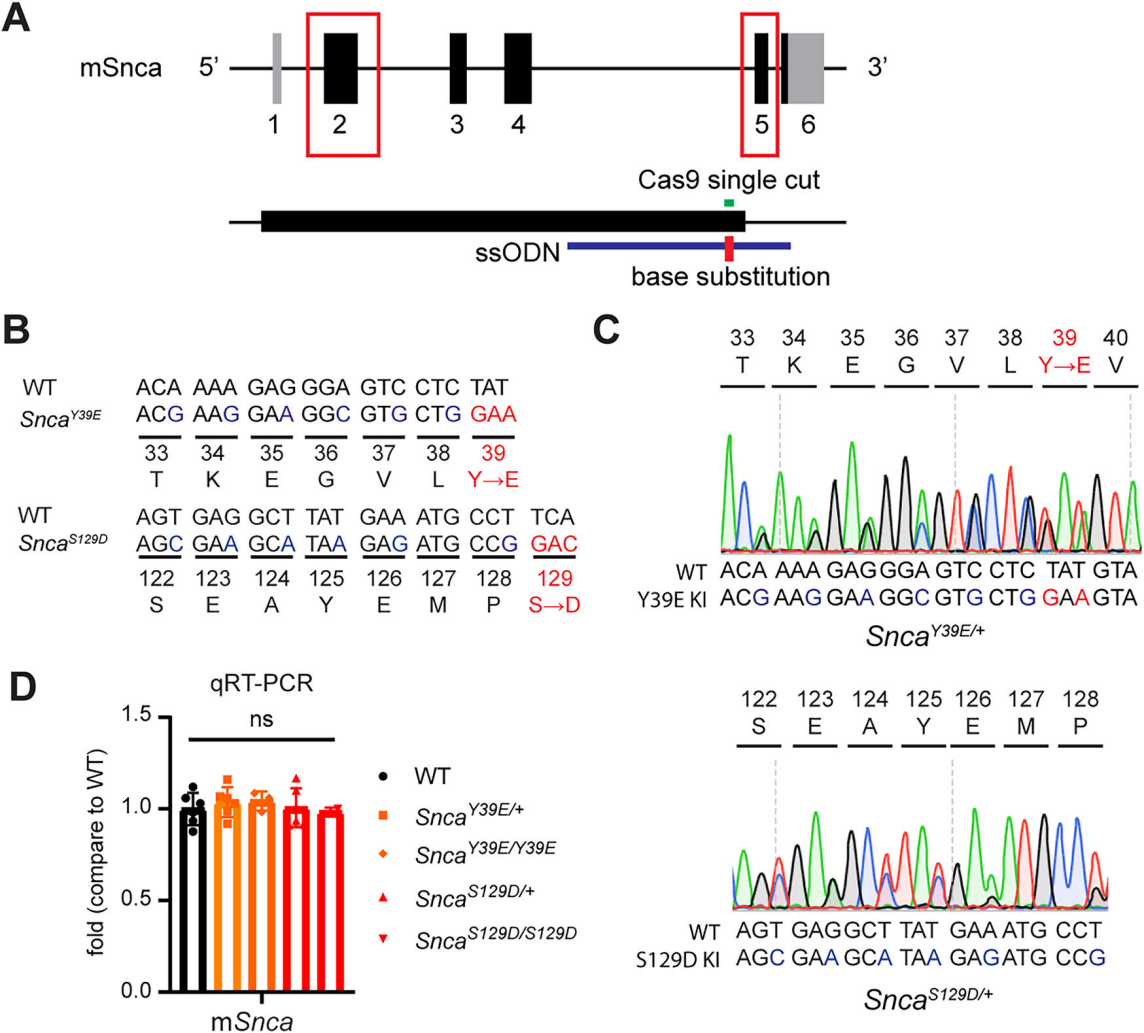
Generation of α-Syn phosphomimetic KI mice. ***A***, Scheme for generation of phosphomimetic mutations of *Snca*. ***B***, Amino acid substitution from original to phosphomimetic mutation. Blue, synonymous mutation for genotyping; red, missense mutation that changes the amino acid to phosphomimetic amino acid. ***C***, Sanger sequencing confirmation with sequence chromatograms for the target sequence of heterozygous Y39E KI mice (top) and heterozygous S129D KI mice (bottom). ***D***, qRT-PCR data for mouse brain *Snca* levels in WT, *Snca^Y39E/+^*, *Snca^Y39E/Y39E^*, *Snca^S129D/+^*, and *Snca^S129D/S129D^* mice. WT, *Snca^Y39E/+^*, and *Snca^S129D/+^* (*n* = 6; *n* = 3 male; *n* = 3 female), *Snca^Y39E/Y39E^* (*n = *3; *n* = 2 for male; *n* = 1 for female), or *Snca^S129D/S129D^* (*n* = 2 for male). ns, not significant. Extended Data Table 1-1 supports Figure 1.

**Figure 2.**
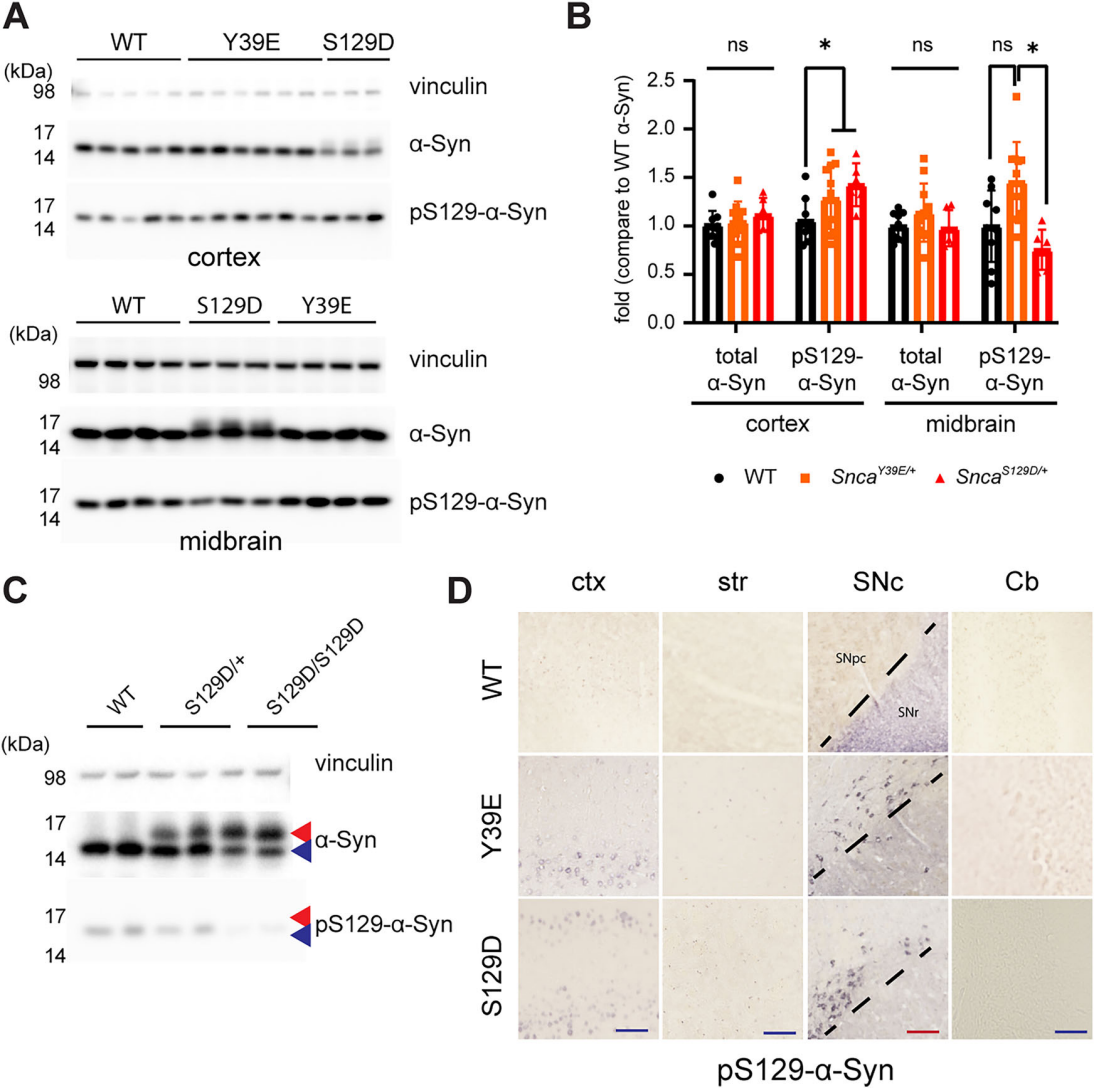
α-Syn phosphomimetic KI mice show increased α-Syn Ser129 phosphorylation in the cortex and substantia nigra. ***A***, Immunoblot image of the cortex (top panel) and midbrain (bottom panel) extracts from 12-month-old WT, *Snca^S129D/+^*, and *Snca^Y39E/+^* mice using antibodies indicated on the right side. ***B***, The relative intensity of immunoblots done using the midbrain extracts of 12-month-old WT, *Snca^Y39E/+^*, and *Snca^S129D/+^* mice for total α-Syn or phospho-S129-α-Syn with vinculin as a loading control. WT (*n* = 9; *n* = 5 male; *n* = 4 female), *Snca^Y39E/+^* (*n* = 11; *n* = 6 male; *n* = 5 female), or *Snca^S129D/+^* (*n* = 6; *n* = 3 male; *n* = 3 female). *p < 0.05; ns, not significant. ***C***, The size-shifted band present in S129D KI mice is S129D mutant-specific. While the normal α-Syn protein migrates at 15 kDa (blue arrowhead), S129D KI mice exhibit a higher molecular weight band (red arrowhead), which is not recognized by the pSer129-α-Syn antibody. ***D***, Representative IHC images of the cortex (ctx), striatum (str), substantia nigra (SNc), and cerebellum (Cb) sections from WT, *Snca^Y39E/+^*, and *Snca^S129D/+^* mice at 12 months labeled with an antibody to detect phospho-S129-α-Syn. Phospho-S129-α-Syn was detected in the cortex and substantia nigra but not in the cerebellum. Scale bar, 250 µm (blue); 150 µm (red).

Among six known phosphorylation sites of the human *SNCA* gene (Y39, S87, Y125, S129, Y133, Y136), we focused on Y39 and S129, which are highly phosphorylated in PD patients (Tenreiro et al., 2014). We introduced the phosphomimetic mutations (Y39E, S129D) into the endogenous mouse *Snca* gene using CRISPR-Cas9 base-exchange technique (Fig. 1*A*; Table 1; see Material and Methods). The correct base substitution in the target genome region was confirmed via genomic PCR and sequencing. Sequencing results confirmed that the mutants had the intended substitution with synonymous mutation for genotyping purposes (Fig. 1*B*,*C*). We observed that the mRNA of *Snca^Y39E/+^* (hereafter Y39E), *Snca^Y39E/Y39E^*, *Snca^S129D/+^* (hereafter, S129D), and *Snca^S129D/S129D^* mice were comparable with that of WT when using qRT-PCR (Fig. 1*D*; Extended Data Table 1-1). Western blot analysis revealed that while total α-Syn levels were similar between WT and KI mice, S129D mice exhibited two distinct bands: one corresponding to endogenous α-Syn, less than molecular weight of 17 kDa, and another above 17 kDa (Fig. 2*A*,*B*). The higher molecular weight band was negative for the pSer129-α-Syn antibody, indicating a subset of the S129D mutant α-Syn undergoing a protein shift (Fig. 2*C*; Extended Data Table 1-1). Phosphorylation of α-Syn at Serine 129 (pS129-α-Syn) is widely recognized as a key marker of α-Syn pathology in PD (Brahmachari et al., 2016). Thus, we examined whether pS129-α-Syn was present and, if so, which brain regions were pS129-α-Syn positive in Y39E and S129D mice. From this analysis at 12 months of age, we observed a robust pS129-α-Syn–positive signal in the cortex and substantia nigra, whereas it was minimal in the striatum, and almost no phosphorylation signal was observed in the cerebellum of Y39E and S129D mice (Fig. 2*D*).

10.1523/ENEURO.0357-24.2025.t1-1 Table 1-1 quantification data for all WB, IF, and IHC images (Fig.1 to Fig. 7). Download Table 1-1, XLS file.

### Y39E and S129D KI mice show decreased membrane-bound α-Syn and increased soluble oligomeric α-Syn species

**Figure 3.**
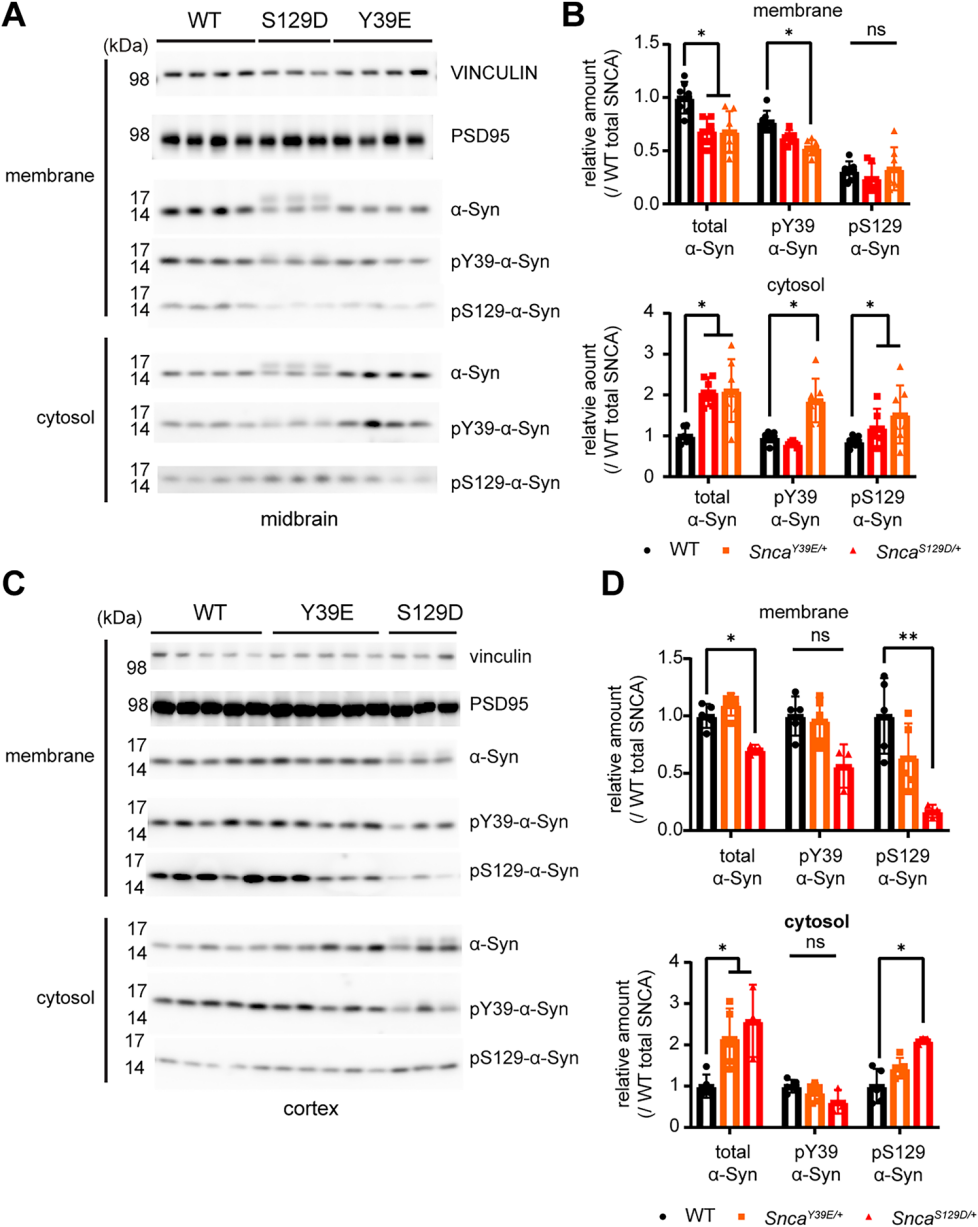
α-Syn is decreased in membrane-enriched fraction of the Y39E and S129D KI mice cortex and midbrain. ***A***, α-Syn distribution in membrane-enriched fraction and a cytosol-enriched fraction of the midbrain and striatum of 12-month-old WT, *Snca^Y39E/+^*, and *Snca^S129D/+^* mice determined using antibodies indicated on the right side. ***B***, Relative amount of total and phospho-α-Syn in membrane-enriched fraction and a cytosol-enriched fraction of midbrain of 12-month-old WT, *Snca^Y39E/+^*, and *Snca^S129D/+^* mice. *Snca^Y39E/+^* and *Snca^S129D/+^* mice show less α-Syn in membrane-enriched fraction and more in cytosol-enriched fraction. WT (*n* = 7; *n* = 4 male; *n* = 3 female), *Snca^Y39E/+^* (*n* = 7; *n* = 4 male; *n* = 3 female), or *Snca^S129D/+^* (*n* = 6; *n* = 3 male; *n* = 3 female). *p < 0.05; ns, not significant. ***C***, Membrane-enriched and cytosol-enriched cortical fractions of 12-month-old WT, *Snca^Y39E/+^*, and *Snca^S129D/+^* mice labeled with total and phospho-α-Syn antibodies. ***D***, Relative amount of total and phospho-α-Syn in membrane-enriched fraction and a cytosol-enriched fraction of cortices of 12-month-old WT, *Snca^Y39E/+^*, and *Snca^S129D/+^* mice. *Snca^Y39E/+^* and *Snca^S129D/+^* mice show less 129 α-Syn in membrane-enriched fraction and more in cytosol-enriched fraction. WT (*n* = 5; *n* = 3 male; *n* = 1 female), *Snca^Y39E/+^* (*n* = 5; *n* = 3 male; *n* = 2 female), or *Snca^S129D/+^* (*n* = 3; *n* = 2 male; *n* = 1 female). *p < 0.05; **p < 0.01; ns, not significant.

**Figure 4.**
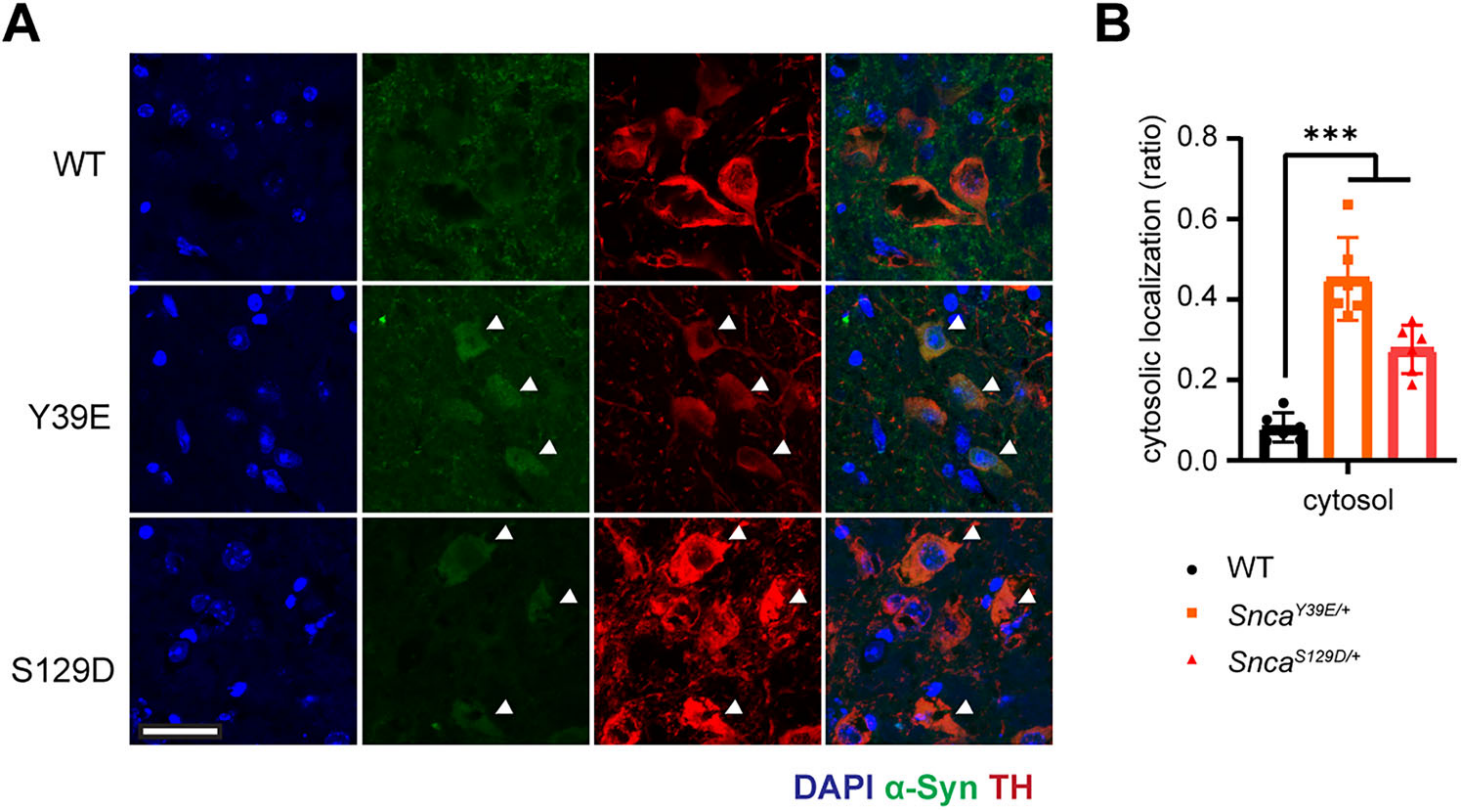
Enhanced cytosolic localization of α-Syn in dopaminergic neurons of Y39E and S129D KI mice. ***A***, Representative IF images depicting the localization of α-Syn within dopaminergic neurons using TH as a marker. *Snca^Y39E/+^* and *Snca^S129D/+^* mice show cytosolic localization of α-Syn in dopaminergic neurons in the substantia nigra. Scale bar (white), 50 µm. ***B***, Quantification for the cytosolic α-Syn colocalization within the TH-positive dopaminergic neurons. WT (*n* = 6; *n* = 3 male; *n* = 3 female), *Snca^Y39E/+^* (*n* = 6; *n* = 3 male; *n* = 3 female), or *Snca^S129D/+^* (*n* = 6; *n* = 3 male; *n* = 3 female). ***p < 0.001.

Previous in vitro, *S. cerevisiae*, and *C. elegans* studies suggested that phosphorylation at Y39 or S129 residue alters the membrane binding of α**-**Syn (Fiske et al., 2011; Kuwahara et al., 2012). To verify this in our mouse models, we performed the membrane and cytosol fractionation of the cortex and midbrain tissue (Nuber et al., 2024). These results showed reduced membrane-bound α-Syn in the midbrain of Y39E and S129D mice and the cortex of S129D mice, with more α-Syn localized to the cytosol of the Y39E and S129D mice cortex and midbrain (Fig. 3*A–D*; Extended Data Table 1-1). Immunostaining results also confirmed more α-Syn localization to cytosol in dopaminergic neurons of Y39E and S129D KI mice (Fig. 4*A*,*B*; Extended Data Table 1-1).

**Figure 5.**
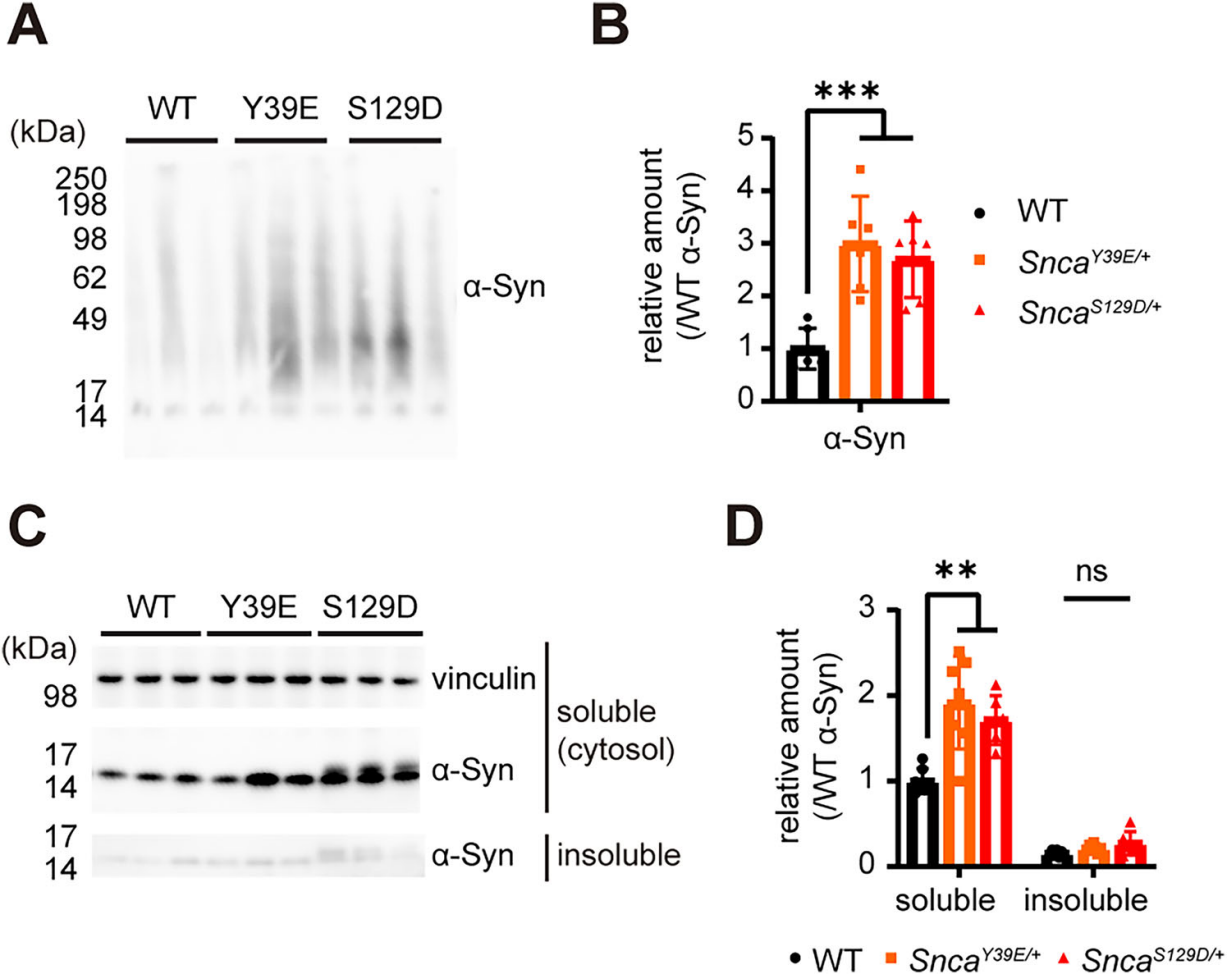
Oligomer and detergent-insoluble aggregates of α-Syn in Y39E and S129D KI mice. ***A***, Blue native polyacrylamide gel electrophoresis image of α-Syn oligomers detected in the midbrain extracts of 12 months WT, *Snca^Y39E/+^*, and *Snca^S129D/+^* mice. Increase of oligomer (39–98 kDa) found in the *Snca^Y39E/+^* and *Snca^S129D/+^* mice midbrain tissue. ***B***, Quantification of α-Syn oligomer protein intensities in the midbrain extracts of 12-month-old WT, *Snca^Y39E/+^*, and *Snca^S129D/+^* mice compared with WT α-Syn levels in each group. *n* = 6; ***p < 0.001. ***C***, Immunoblot image of soluble and 1% Triton X-100 insoluble α-Syn from midbrain extracts of WT, *Snca^Y39E/+^*, and *Snca^S129D/+^* mice with vinculin as loading control and antibodies indicated on the right side. ***D***, Quantification of soluble and 1% Triton X-100 insoluble α-Syn compared with WT α-Syn levels. WT (*n* = 7; *n* = 4 male; *n* = 3 female), *Snca^Y39E/+^* (*n* = 7; *n* = 4 male; *n* = 3 female), or *Snca^S129D/+^* (*n* = 6; *n* = 3 male; *n* = 3 female). *p < 0.05; ns, not significant.

It is shown that phosphorylated α-Syn enhance forming toxic soluble oligomeric species (Bengoa-Vergniory et al., 2017; Emin et al., 2022) and insoluble aggregates (Wang et al., 2019, Suzuki et al., 2020). To further examine whether pathologic α-Syn species are formed in Y39E and S129D KI mice, we measured the oligomeric species of α-Syn from Y39E and S129D mice by running a native gel (BN-PAGE). Brain extract of 12-month-old Y39E and S129D KI mice showed soluble oligomeric species of α-Syn as indicated by molecular weight over 17 kDa to higher than 98 kDa in BN-PAGE analysis (Fig. 5*A*,*B*; Extended Data Table 1-1). Next, we measured the detergent-insoluble α-Syn aggregates by serial fractionation using Triton X-100 and SDS, and we found that there was no difference in the level of Triton X-100 insoluble α-Syn between WT and KI mice lines at 12 months of age (Fig. 5*C*,*D*; Extended Data Table 1-1). These data indicate that Y39E and S129D KI mice form pathological species of α-Syn including the phosphorylated and soluble oligomeric species.

### Y39E and S129D KI mice do not show astrogliosis, microgliosis, or neurodegeneration in the striatum and substantia nigra

**Figure 6.**
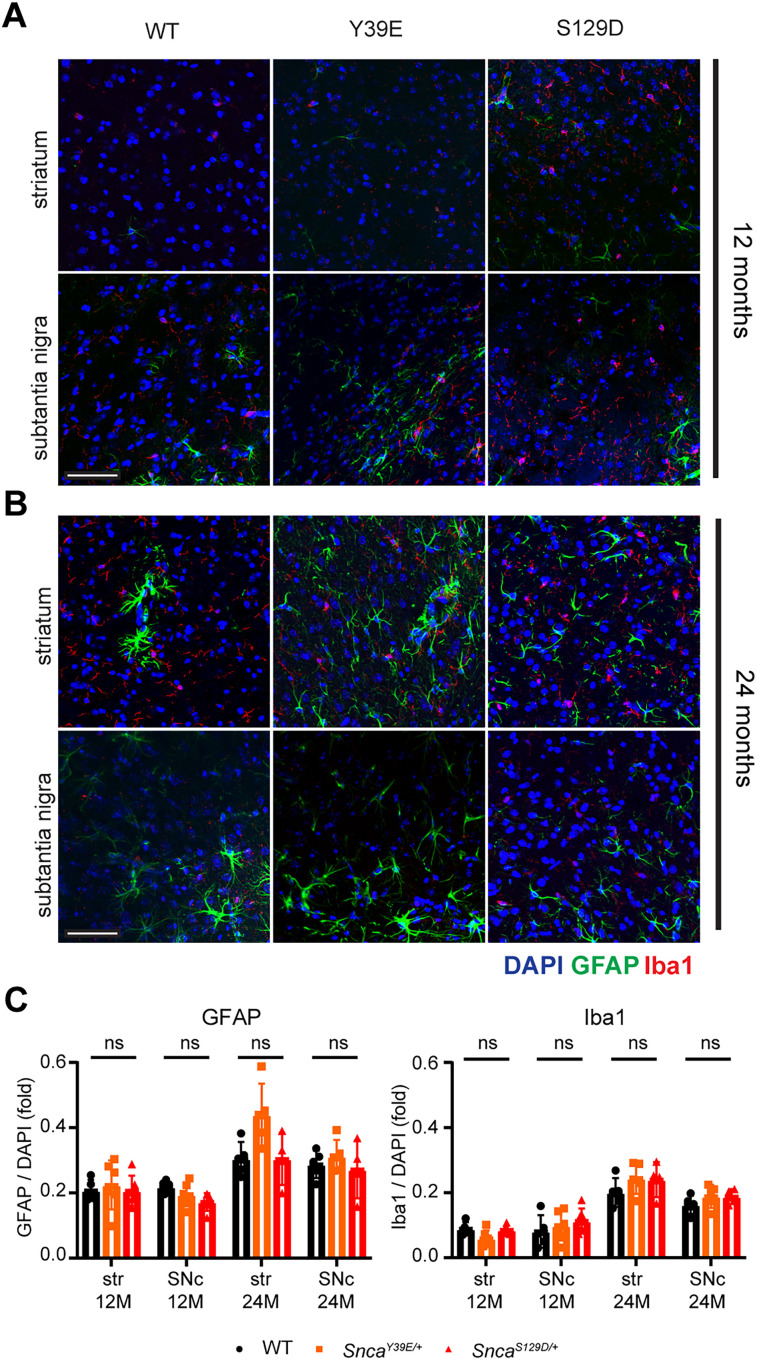
Astrogliosis and microgliosis were not observed in Y39E and S129D KI mice. Glial activation was assessed in the cryopreserved sections of the substantia nigra and striatum from 12- and 24-month-old WT, *Snca^Y39E/+^*, and *Snca^S129D/+^* mice (*n* = 3). ***A***, Representative images of astrocyte and microglia activation in the striatum and substantia nigra of 12-month-old WT, *Snca^Y39E/+^*, and *Snca^S129D/+^* mice visualized with GFAP and Iba1 double immunostaining. Scale bar (white), 50 µm. ***B***, Representative images of astrocyte and microglia activation in the striatum and substantia nigra of 24-month-old WT, *Snca^Y39E/+^*, and *Snca^S129D/+^* mice visualized with GFAP and Iba1 double immunostaining. Scale bar (white), 50 µm. ***C***, Relative quantities of GFAP and Iba1 intensities compared with DAPI. Twelve-month-old WT (*n* = 5; *n* = 3 male; *n* = 2 female), 12-month-old *Snca^Y39E/+^* (*n* = 6; *n* = 3 male; *n* = 3 female), or 12-month-old *Snca^S129D/+^* (*n* = 5; *n* = 3 male; *n* = 2 female). Twenty-four-month-old WT (*n* = 5; *n* = 3 male; *n* = 2 female), 24-month-old *Snca^Y39E/+^* (*n* = 5; *n* = 3 male; *n* = 2 female), or 24-month-old *Snca^S129D/+^* (*n* = 4; *n* = 2 male; *n* = 2 female). ns, not significant.

Astrogliosis (Liddelow et al., 2017; Chun and Lee, 2018; Zeng et al., 2020) and microglia activation (McGeer et al., 1988; Hong et al., 2016) are common features in PD and various other neurodegenerative diseases. To investigate whether Y39E and S129D mice exhibit neuroinflammation, we assessed markers of astrogliosis (GFAP) and microgliosis (Iba1) in the striatum and substantia nigra. No significant differences were observed at 12 or 24 months of age (Fig. 6*A*,*C*; Extended Data Table 1-1).

**Figure 7.**
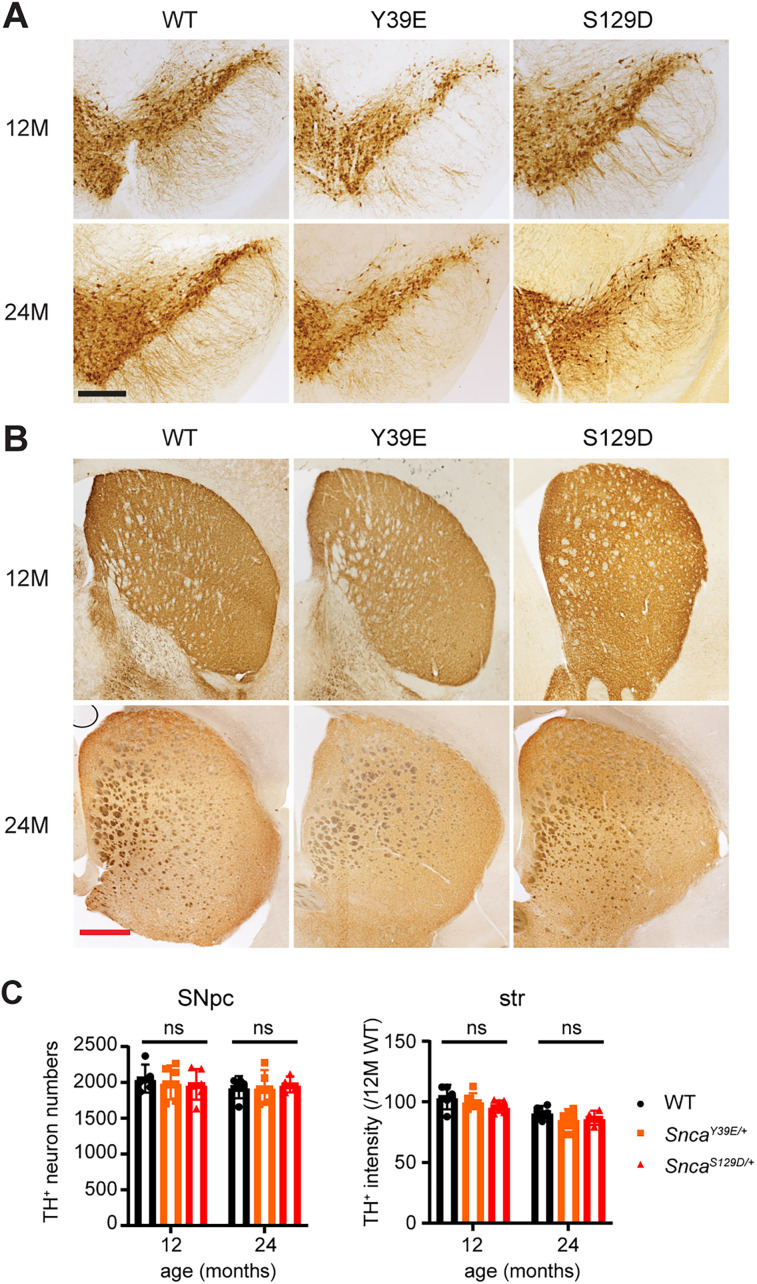
Dopaminergic neuron numbers in Y39E and S129D KI mice remained unaltered up to 24 months of age. ***A***, Representative image of TH-positive dopaminergic neurons in the substantia nigra of 12- and 24-month-old WT, *Snca^Y39E/+^*, and *Snca^S129D/+^* mice. Scale bar (black), 400 µm. ***B***, Representative image of TH-positive dopaminergic neurites in the striatum of 12- and 24-month-old WT, *Snca^Y39E/+^*, and *Snca^S129D/+^* mice. Scale bar (red), 500 µm. ***C***, Quantification of a TH-positive cell number in the substantia nigra (left panel) and relative intensities of TH-positive signal in the striatum (right panel) from cryopreserved sections of 12- and 24-month-old WT, *Snca^Y39E/+^*, and *Snca^S129D/+^* mice. Twelve-month-old WT (*n* = 5; *n* = 3 male; *n* = 2 female), 12-month-old *Snca^Y39E/+^* (*n* = 6; *n* = 3 male; *n* = 3 female), or 12-month-old *Snca^S129D/+^* (*n* = 5; *n* = 3 male; *n* = 2 female). Twenty-four-month-old WT (*n* = 5; *n* = 3 male; *n* = 2 female), 24-month-old *Snca^Y39E/+^* (*n* = 5; *n* = 3 male; *n* = 2 female), or 24-month-old *Snca^S129D/+^* (*n* = 4; *n* = 2 male; *n* = 2 female). ns, not significant.

In PD patients and α-Syn mouse models, the substantia nigra exhibits dopamine neuronal loss and reduction of dopaminergic neurons neurites in the striatum (Lo Bianco et al., 2002, Surmeier et al., 2017). To determine whether Y39E and S129D mice show degeneration of dopaminergic neurons in the striatum and substantia nigra, we estimated the level of dopaminergic neurons by measuring TH-positive intensity (striatum) and cell numbers (substantia nigra). Y39E and S129D did not show loss of TH-positive dopaminergic neurons in the substantia nigra or reduction in dopaminergic neuronal fibers in the striatum up to 24 months of age (Fig. 7*A*–*C*; Extended Data Table 1-1). These results suggest that Y39E and S129D mice do not exhibit significant neuroinflammation or neurodegeneration.

### Y39E and S129D KI mice do not show motor-deficit phenotypes up to 12 months of age

**Figure 8.**
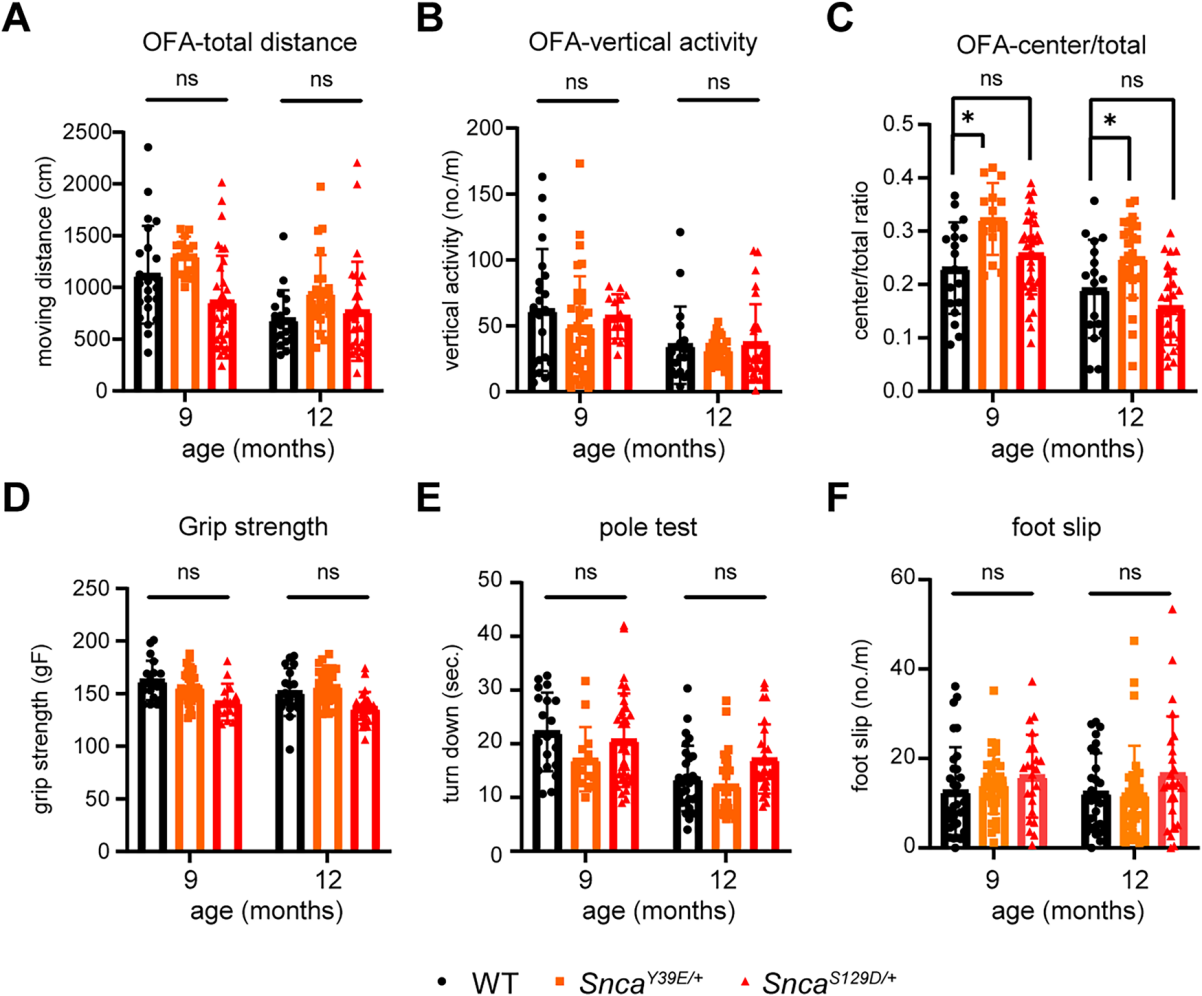
No motor functional deficits were observed in Y39E and S129D mice up to 12 months of age. We compared the motor function of *Snca^Y39E/+^* and *Snca^S129D/+^* KI mouse lines to WT littermates at 9 and 12 months of age. ***A***, General mouse activity was measured with total distance traveled in the open-field test. ***B***,***C***, Anxiety behavior was analyzed by vertical activity **(*B*)** and ratio of movement in the central zone (***C***) of open-field activity. *Snca^Y39E/+^* spends more time in the center area than its WT littermates in 9 and 12 months. ***D***,***E***, Motor strength was assessed by grip strength (***D***) and the pole-hanging test (***E***). ***F***, Motor coordination was measured by the number of foot slips on the parallel rod floor test. WT (*n* = 32; *n* = 16 male; *n* = 16 female), *Snca^Y39E/+^* (*n* = 28; *n* = 14 male; *n* = 14 female), *Snca^S129D/+^* (*n* = 23; *n* = 11 male; *n* = 12 female). *p < 0.05, ns, not significant.

Considering that motor function decline is one of the clinical symptoms of PD, we evaluated the motor function using various behavioral tests. In the open-field activity test, which measures locomotor activity and anxiety-related behavior, neither KI mouse line showed differences in movement, distance traveled, or vertical activity (Fig. 8*A*,*B*). However, Y39E mice showed a mild but significant increase in central/total distance ratio, representing less anxiety-like behavior at 9 and 12 months of age (Fig. 8*C*). Additional motor behavioral assays, including the pole test, grip strength test, and foot slip measured by parallel rod floor test, revealed no differences in muscle strength (pole test, grip strength test) or motor coordination (parallel rod floor test) between WT and KI mice (Fig. 8*D*–*F*). Taken together, these data indicate that Y39E and S129D mice do not show motor phenotypes seen in PD patients up to 12 months of age.

## Discussion

In this study, we generated KI mouse models expressing Snca phosphomimetic mutants Y39E and S129D, known phosphorylation sites in α-Syn associated with PD and other synucleinopathies. These mutations were expressed under the endogenous promoter to mimic the spatiotemporal pattern of mouse *Snca* gene expression. The KI mouse models exhibit progressive α-Syn phosphorylation, reduced membrane localization, and elevated levels of soluble oligomers of α-Syn. However, they did not develop dopaminergic neuronal degeneration or motor behavioral impairments typically related to PD pathology.

Previous mouse models investigating α-Syn phosphorylation used the knock-out of kinases that target α-Syn phosphorylation sites (C-Abl, PLK2; Brahmachari et al., 2016, Weston et al., 2021) or treatment with kinase inhibitors (Wu et al., 2020). However, these kinases have multiple off-targets other than α-Syn, potentially affecting other molecular pathways. Moreover, overexpression of the S129D- or S129A-α-Syn mutants in different mouse models induced PD-like pathology primarily driven by the protein’s expression level rather than changes in its phosphorylation status (McFarland et al., 2009, Escobar et al., 2014). It is known that duplication or triplication of the *SNCA* gene accelerates the onset and progression of PD symptoms (Singleton et al., 2003), and even nonmutated WT α-Syn can cause dopamine neuronal cell death or PD-like motor deficits depending on its expression level (St Martin et al., 2007; Oliveras-Salvá et al., 2013; Richter et al., 2023). These findings suggest that high expression of α-Syn may obscure the effects of phosphorylation, highlighting the need for appropriate expression levels of phosphomimetic α-Syn mutations to study the role of phosphorylation in the mouse model.

The advantage of using a KI mouse model is that the modified gene is expressed in a manner that is analogous to the spatial and temporal expression of the endogenous gene. To investigate the role of phosphorylation in neurodegenerative disease-associated proteins, several phosphomimetic and phospho-defective mouse models have been developed to validate the in vitro findings. For instance, phosphomimetic APP KI mice (APP^S675D^) showed the enhanced cleavage of endogenous APP into Aβ (Menon et al., 2019), while phosphor mimetic (MAPT^9E18^) and defective (MAPT^9A18^) tau KI mice show altered membrane–cytosol localization (Gilley et al., 2016). More recently, a study using the α-Syn^S129A^ KI mice model revealed that the Serine 129 phosphorylation of α-Syn plays a physiological role in fine-tuning the balance between excitatory and inhibitory neuronal currents, without complication of overexpression (Ramalingam et al., 2023).

Consistent with previous KI mice models, Our Y39E and S129D KI models show expression of phosphomimetic mutant α-Syn protein levels comparable with endogenous WT protein and successfully recapitulated the in vitro findings regarding the role of α-Syn phosphorylation in membrane binding. Notably, both Y39E and S129D KI mice exhibited significant shifts in the localization of α-Syn from the membrane to the cytosol, along with increased levels of soluble α-Syn oligomers, consistent with previous studies. Interestingly, Y39E mice show elevated levels of pY39-α-Syn in the nonmutated, endogenous α-Syn within the cytosol-enriched fraction, suggesting that phosphomimetic mutants might enhance the phosphorylation propensity of nonmutated α-Syn, promoting its detachment from the membrane to the cytosol and enhancing oligomerization.

As the Y39E and S129D KI mice did not exhibit motor deficits up to 12 months of age or a significant reduction in dopaminergic neurons up to 24 months, these findings imply that a single copy of the phosphomimetic mutations may not be sufficient to induce the inflammation, motor deficits, or dopaminergic neuron loss within the study's time frame. However, α-Syn phosphorylation plays a crucial role in modulating excitatory neuronal function (Ramalingam et al., 2023), employing homozygous KI mice might obscure whether the motor-deficit or neuronal change is based on the depletion of endogenous α-Syn phosphorylation. In contrast, using heterozygous KI mice to preserve WT α-Syn function while enabling assessment of phosphomimetic mutations’ effect provides a more physiologically relevant model for studying α-Syn phosphorylation in PD and related synucleinopathies.

In conclusion, our Y39E and S129D KI mice represent novel models that demonstrate the impact of phosphorylation on altered α-Syn localization and soluble oligomer formation, as predicted by in vitro studies. While these KI mouse models did not fully recapitulate all PD-related symptoms and pathology, they serve as valuable tools for further in vivo exploration of the role of α-Syn phosphorylation and membrane binding in neurodegenerative processes.

## Synthesis

Reviewing Editor: Arvind Kumar, KTH Royal Institute of Technology Decisions are customarily a result of the Reviewing Editor and the peer reviewers coming together and discussing their recommendations until a consensus is reached. When revisions are invited, a fact-based synthesis statement explaining their decision and outlining what is needed to prepare a revision will be listed below. The following reviewer(s) agreed to reveal their identity: Jie Shen.

SYNTHESIS

Your manuscript was reviewed by one reviewer. One of the reviewers dropped out after agreeing. We decided to go ahead with one review only in order not to delay the process.

The reviewer has appreciated the work and finds the new KnockOut model may be useful to the community. However, she/he has raised concerns over the experimental design and small sample size. We will be happy to reconsider your manuscript after you have improved on these two crucial aspects. Detailed comments are appended below for you to revise the manuscript.

Reviewer #1

The manuscript is clearly written, and the Y39E and S129D KI mice may be useful for the PD field. The authors convincingly showed that there is an additional a-Syn band in S129D/+ brains, though they did not confirm the identity of the band. However, the small sample size (n=3 for most experiments), the quality of the data in some figures (e.g. Fig. 1F, without quantification), and the experimental design (without using homozygous KI) all made this reviewer less sure about their conclusions. I would suggest the following.

1) use more than 3-4 mice per genotype for key experiments.

2) Fig. 1B: show the sequencing results of WT and KI/KI genomic DNA, instead of hypothetical DNA sequences.

3) both KI lines are newly generated mice, and no Northern blotting data are presented to ensure Snca mRNA is not affected. The qRT-PCR results are quite variable. Minimally they should make the mRNA analysis in KI mice more convincing and KI/KI mice should be used instead of KI/+ mice.

4) Fig. 1D/E: the authors stated that the upper band represents the S129D without experimental data to support this. They should use KI/KI brains to see if the lower band disappears in S129D KI/KI brains and whether there is no band while using pS129-a-Syn antibodies. They don't need to use mibrains for WB and should include cortices in the WB, so they can compare the WB results with immunostaining data.

5) Fig. 1F: Based on WB data in Fig. 1D, pS129-a-Syn levels are reduced in S129D KI/+ brains (midbrains used), but immunostaining data showed elevated pS129-a-Syn immunoreactivity in the cortex of S129D KI/+ mice. Better characterization of both KI mice will make the paper stronger and make the mice more valuable to other researchers in the field.

6) Figure 2 leads to a major conclusion of the paper but similar problems exist, making it hard to draw these conclusions. The authors just need to include KI/KI, use both cortex and ventral midbrains as well as a larger n. It seems that they only quantified 4 TH+ neurons, whereas they should have used at least 10 neurons per mouse, minimally 4-5 mice per genotype.
